# ZIKV Infection Induces DNA Damage Response and Alters the Proteome of Gastrointestinal Cells

**DOI:** 10.3390/v12070771

**Published:** 2020-07-17

**Authors:** Kathleen Glover, Kevin M. Coombs

**Affiliations:** 1 Department of Medical Microbiology and Infectious Diseases, Manitoba Centre for Proteomics & Systems Biology, Room 799, University of Manitoba, 715 McDermot Avenue, Winnipeg, MB R3E 3P4, Canada; gloverk@myumanitoba.ca; 2 Children’s Hospital Research Institute of Manitoba, Room 513, John Buhler Research Centre, 715 McDermot Avenue, Winnipeg, MB R3E 3P4, Canada

**Keywords:** flavivirus, dysregulated proteins, bioinformatics, gastrointestinal disease, proteomics, aptamers, SomaScan

## Abstract

The zika virus (ZIKV) is a neurotropic virus that causes congenital abnormalities in babies when they are infected in utero. Some studies have reported these congenital abnormalities result from ZIKV attacking neural progenitor cells within the brain which differentiate into neurons, oligodendrocytes, and astrocytes. Each of these glial cells play important roles during development of the fetal brain. In addition to ZIKV-induced congenital abnormalities, infected patients experience gastrointestinal complications. There are presently no reports investigating the role of this virus at the proteomic level in gastrointestinal associated cells, so we conducted an in vitro proteomic study of ZIKV-induced changes in Caco-2, a colon-derived human cell line which is known to be permissive to ZIKV infection. We used SomaScan, a new aptamer-based proteomic tool to identify host proteins that are dysregulated during ZIKV infection at 12, 24, and 48 h post-infection. Bioinformatic analyses predicted that dysregulation of differentially-regulated host proteins results in various gastrointestinal diseases. Validation of the clinical relevance of these promising protein targets will add to the existing knowledge of ZIKV biology. These potential proteins may be useful targets towards the development of therapeutic interventions.

## 1. Introduction

Zika virus (ZIKV) is a neurotropic flavivirus. ZIKV has been recognized for several decades, and causes serious clinical manifestations, but there still are no FDA approved therapeutic interventions against it [1,2]. More than two years after WHO declared ZIKV a public health threat, global ZIKV prevalence dropped drastically [3]. Despite this drastic decline in global prevalence, there remains a need to develop effective therapeutic interventions against ZIKV and other arbovirus infections. This will mitigate possible future epidemics and pandemics, as almost occurred during the 2014–2016 Ebola virus outbreak, which resulted in thousands of deaths in various countries due to the absence of an effective vaccine [4]. Several factors which might result in the occurrence of a potential ZIKV pandemic include changes in the virulence of this virus, increased global travel and changes in climate conditions leading to geographic spread of the Aedes mosquito species [5].

Clinical symptoms of ZIKV infection often mirror those induced by Dengue virus (DENV), another flavivirus. Thus, patients are often misdiagnosed [6]. In addition to DENV-like symptoms, reports of gastrointestinal disturbance in ZIKV-infected patients have been reported. These include nausea, abdominal pain, diarrhea, and vomiting [7,8,9,10,11]. So far, ZIKV gastrointestinal involvement, as measured at the proteomic level, has not been reported.

We performed extensive discovery-based proteomic analyses of ZIKV-infected Caco-2 cells using the aptamer-based SomaScan. Caco-2 are human colorectal adenocarcinoma cells derived from the gastrointestinal tract [12]. These cells were chosen since gastrointestinal tract complications have been reported to arise during ZIKV infection [13]. These cells are highly permissive to ZIKV infection [14]. Caco-2 cells were infected with ZIKV and analyzed at 12, 24 and 48 h post-infection (hpi) to determine changes within the cellular proteome after infection. Changes within the proteome were identified by comparing the expression profiles of host proteins identified after ZIKV infection to their corresponding time-matched non-infected mock controls. A total of 446 host proteins were significantly dysregulated at any time point, and 71 were dysregulated > 1.375-fold (Table 1). The majority of dysregulated proteins were significantly upregulated at 12 hpi, irrespective of the fold-change cut-off used (Table 1), a pattern that was not reflected by our previous analyses of ZIKV-infected Vero [15], U-251 astrocytoma [16] or Sertoli cells [17]. Bioinformatic analysis using Ingenuity Pathway analysis (IPA) predicted several pathways that were activated and inhibited by ZIKV infection. Pathways predicted to be activated or inhibited were CDK5 signaling, neuroinflammation signaling, dendritic cell maturation, FGF signaling, and G2/M DNA damage checkpoint regulation. Gastrointestinal diseases were among the top diseases and functions identified by IPA. Further studies using a suitable in vivo model need to determine how host proteins identified from our protein screen result in gastrointestinal complications already reported in ZIKV-infected patients. These gastrointestinal-associated host proteins may represent other promising anti-viral targets.

## 2. Materials and Methods

Infections, sample processing for SomaScan analyses, plaque titrations in Vero cells (ATCC^®^ Number: CCL-81^™^, Manassas, VA, U.S.A.), immunofluorescence, cell viability assays, immunoblotting and bioinformatics were performed essentially as described [15], with some modifications. Briefly, Caco-2 (ATCC^®^ Number: HTB-37^™^) infections were optimized by comparing virus yields, cell cytopathology and efficiency of cell infections under various multiplicity of infection (MOI) parameters. For proteomic and immunoblotting analyses, cells were grown to ~80% confluency, infected with ZIKV using an MOI of 3, and cells were harvested after 12, 24, and 48 h of ZIKV infection. Thirty μg of various cell lysates were resolved in SDS-PAGE and probed with α-ZIKV NS-1 Ab (GeneTex cat # GTX133307; Irvine, CA, U.S.A.) to confirm infections. Seventy (70) µL of 200 µg/mL protein in every cell lysate was prepared and submitted for SomaScan analysis on our in-house Soma Logics^®^ (Boulder, CO, U.S.A.)-licensed platform at the Manitoba Centre for Proteomics and Systems Biology. The SomaScan analyses were performed on three separate biologic replicates of infected and time-matched mock-infected samples (18 total samples). Relative fluorescent units (RFU) were determined for each of 1305 proteins in each sample and converted to Log_2_ values. Differences between each infected sample and its time-matched mock, non-infected sample were examined by Student’s T-test and by Z-score. Calculated fold changes (using cut-offs of > ± 1.3-fold and *p*-values < 0.05) were imported into Ingenuity Pathway Analysis (IPA; Qiagen, Hilden, Germany) software to identify cellular pathways, top disease and biofunctions affected by ZIKV. STRING (https://string-db.org) protein-protein interaction network functional enrichment analysis was used to identify interactions between host proteins which were commonly and differentially dysregulated in Caco-2 cells. Representation of all graphs and volcano plots was performed using GraphPad Prism 6.0 (San Diego, CA, U.S.A.) or SigmaPlot 11.0 (Santa Clara, CA, U.S.A.) software. 

## 3. Results

### 3.1. ZIKV Virus Induces Cytopathology in Caco-2 with Increased Viral Titer

**Figure 1:**
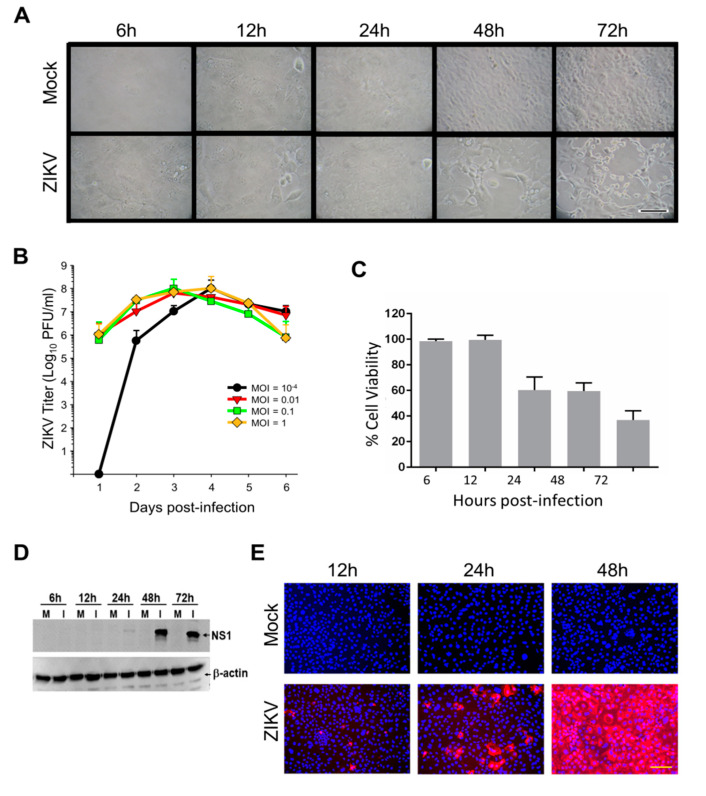
ZIKV growth kinetics in Caco-2 cells and proteomic validation. (**A**) Photomicrographs of Mock and Zika virus (ZIKV) infected Caco-2 cells at indicated times post-infection. (**B**) Kinetics of virus production after different MOI infections. Error bars represent S.E.M. of three replicates. (**C**) Cytopathology induced at different times post-infection, determined by WST-1 cell viability. Error bars represent S.E.M. of three replicates. (**D**) ZIKV non-structural protein-1 (NS1) expression in mock-infected (M) and in virus-infected (I) cells. (**E**) Immunofluoresence staining showing expression of ZIKV NS1 protein (red) as a function of time after ZIKV MOI = 3 infection. Nuclei were stained with DAPI (blue). Scale bars in **A** and **E**, (lower right micrographs) represent 100 µm.

ZIKV-induced cytopathic effects (CPE) were noticeable by 24 hpi and were more pronounced after 48 hpi (Figure 1A). ZIKV growth curves also were performed to confirm our cells would support ZIKV replication and to determine appropriate time points for subsequent analyses (Figure 1B). Peak titers exceeded 10^8^ PFU/mL by days 3 and 4, even when cultures were infected at multipli-cities of infection (MOI) < 0.01. We then infected cells at an MOI of 3, predicted by Poisson distribution to result in >95% initial cell infection, as done in our previous ZIKV proteomic studies [15].

Simultaneously, a WST-1 (4-[3-(4-iodophenyl)-2-(4-nitrophenyl)-2*H*-5-tetrazolio]-1, 3-benzene disulfonate; Pierce Biotechnology) cytotoxicity assay was performed to more precisely measure Caco-2 cell viabilities at various times after ZIKV infection. We observed no CPE up to and including 24 hpi, but CPE was apparent, and increased from ~50% at 48 hpi to ~70% by 72 hpi (Figure 1C). ZIKV NS1 expression was examined; faint immunoreactive bands were observed at 24 hpi and signal was significantly stronger by later time points (Figure 1D). Immunofluorescence microscopy indicated that virtually every cell was infected by 48 hpi (Figure 1E), confirming that an MOI = 3 successfully infected every cell. Based on these cumulative data, we chose 12, 24 and 48 hpi as time points to probe by SomaScan.

### 3.2. ZIKV Induces Proteomics Dysregulation of Caco-2 Host Proteins

**Figure 2:**
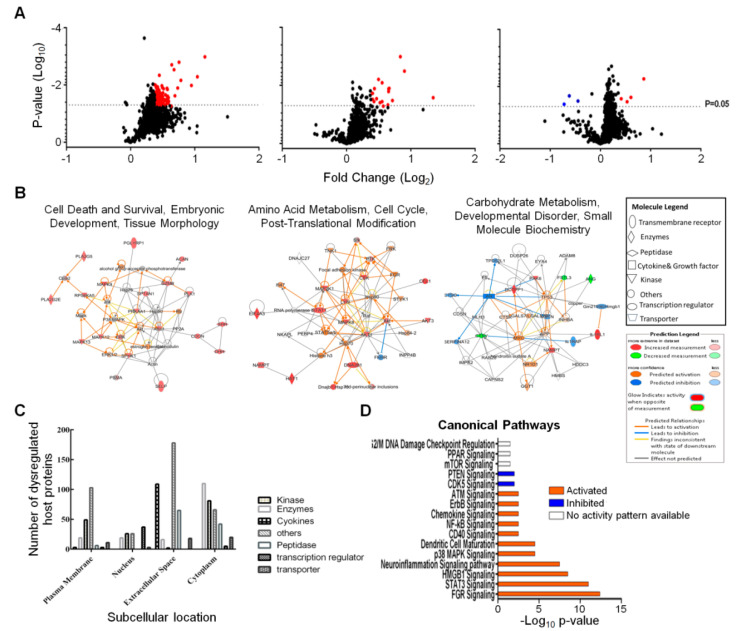
Zika virus dysregulated Caco-2 proteins and predicted signaling pathway. (**A**) Volcano plots showing fold changes and *p*-values of Caco-2 proteins at each time point. Red dots are significantly upregulated proteins. The three blue dots in the 48 h plot are significantly downregulated. (**B**) IPA-determined interaction networks and focus molecules of top diseases and functions predicted to be affected after ZIKV infection. Orange proteins are upregulated, whereas downregulated proteins are green. Predicted pathway activations and inhibitions are depicted in orange and blue, respectively. (**C**) Subcellular locations of dysregulated host proteins and the nature of these proteins. (**D**) Bar charts showing signaling pathways predicted by IPA to be induced after 12 h of ZIKV infection. Orange bar and blue bars indicates activation and inhibition of pathways after infection, respectively. Clear bars indicate no activity prediction by IPA based on Z score.

We screened and measured dysregulation of 1305 Caco-2 proteins in triplicate from three different time points using the aptamer-based SomaScan proteomic tool. Statistical analyses, using both Student’s T-test and Z-score, identified 439 proteins that were significantly dysregulated at any time point (Table 1). The vast majority of these were upregulated at 12 hpi. More than 100 proteins were also significantly dysregulated at 48 hpi and virtually all of these also were upregulated. We routinely apply more stringent fold-change cut-off criteria to such lists of proteins [15]. A total of 193 proteins were significantly dysregulated ≥ 1.30-fold (= ≤ 0.7693-fold if downregulated) and these are depicted in Figure 2A and were imported into IPA for bioinformatics analyses. Table 2 displays the 71 total Caco-2 proteins that were significantly dysregulated ≥ 1.375-fold (= ≤ 0.7273-fold if downregulated) across all three time points. Of the 71 proteins dysregulated, ≥ 1.375-fold, 52 were upregulated at 12 hpi, 15 were upregulated at 24 hpi, 2 were upregulated at 48 hpi and only 2 were downregulated, and only at 48 hpi.

The entire dataset was imported into IPA for analysis. Figure 2B displays networks of the top Diseases and Functions with a score of >30 and >20 focus molecules. The most significantly affected networks at 12 hpi were cell death and survival, embryonic development, tissue morphology, amino acid metabolism, cell cycle, post-translational modification at 24 hpi and carbohydrate metabolism, developmental disorder, small molecule biochemistry at 48 hpi. Each network at each time point was overlaid with proteomic data from the other time points to visualize changes in expression profiles of the individual proteins in each network over time. Most of these significantly dysregulated proteins represented kinases, enzymes, cytokines and other molecules predicted to reside in various subcellular compartments (Figure 2C). Most of the dysregulated host proteins were classified as “others” and were located in the “extracellular space”.

IPA predicted several signaling pathways and linked various cellular processes that were activated or inhibited after 12 h of ZIKV infection (Figure 2D). Activation and inhibition of each pathway was based on positive or negative Z-scores. Pathways that were linked to immunity included dendritic cell maturation, STAT3, chemokine, CD40, NF-kB and p38 MAPK signaling [18,19,20,21,22]. Other pathways, such as HMGB1, CDK5, ATM and G2/M DNA damage checkpoint regulation, neuroinflammation and ErbB signaling are all linked to various cell cycle processes [23,24,25,26,27,28]. 

### 3.3. ZIKV Infection Results in Numerous Diseases and Alters Biofunctions

**Figure 3:**
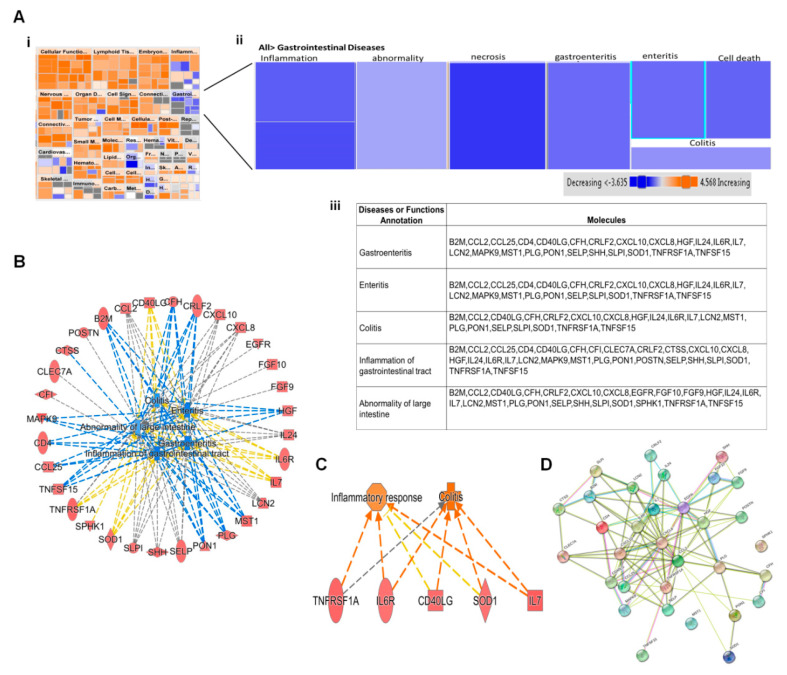
Proteomic prediction by IPA of Disease and function affected by ZIKV infection. (**A**) (**i**) and (**ii**) Heatmaps of various diseases and functions after 12 h of ZIKV infection. Among various disease and function predicted are various forms of gastrointestinal diseases. Predictions of activation of various disease and function were based on Z scores. Orange color represents activation; blue indicates inhibition. (**iii**) Tabulation of the various dysregulated host processes which are linked to various gastrointestinal diseases. (**B**) Protein-protein interaction among proteins in Figure 3iii generated by String software. (**C**) Networks of dysregulated host proteins linked to the various gastrointestinal diseases predicted by IPA. (**D**) Network of predicted effect of increased expression of downstream molecules involved in gastroenteritis.

Bioinformatic analysis by IPA predicted induction of several diseases and altered biofunctions as a result of ZIKV infection. Most of these alterations were observed at 12 hpi since more than 80% of host proteins dysregulated were at this early time point. Activation of all diseases and function were based on their Z-scores. Diseases and functions with Z-score ≥ 2.0 were predicted to have increased activation while those with Z-score ≤ −2.0 have decreased activation (Figure 3Ai). Biofunctions whose activations were predicted to be either increased or decreased included cell movement of dendritic cells, binding of T Lymphocytes, chemotaxis of neutrophils, quantity of antigen presenting cells and inflammation of body cavity. Previous ZIKV proteomic studies had identified development and quality of neurons, development of sensory organ, sensory system development and synthesis of lipid [15,16]. Proteomic delineation of gastrointestinal complications induced by ZIKV has not been reported yet. Among the gastrointestinal diseases and biofunctions predicted to be activated by ZIKV are gastroenteritis, enteritis, colitis, inflammation of gastrointestinal tract and abnormality of large intestine (Figure 3Aii). IPA predicted a significant activation of all gastrointestinal complication based on their *p*-values of < 0.05, but no predicted activation due to Z-score of ≤ −2.0. All these predictions were induced by >20 dysregulated host proteins which were significantly up regulated at 12 hpi. Host proteins that were commonly linked to all the predicted gastrointestinal complications were cytokines (CCL2, CCL25), chemokines (CXCL10, CXCL8 and CRLF2), interleukins (IL24, IL6R and IL7), complement factor H, tumor necrosis factors (TNFRSF1A, TNFSF15), T-cell interacting proteins (CD40LG), and MAP kinase 9 (Figure 3Aiii). Other uniquely expressed proteins included FGF9 and FGF10, EGFR, CFI, POSTN, 

CTSS, SPHK1 and CLEC7A. Figure 3B displays the protein-protein interactions between all the proteins in Figure 3Aiii as determined by STRING analysis. All proteins except SPHK1 and MST1 interact with each other. We have similarly explored interacting significantly dysregulated host proteins during influenza a virus infection [29]. 

### 3.4. Proteomic Prediction of ZIKV Activation of DNA Damage Response

**Figure 4:**
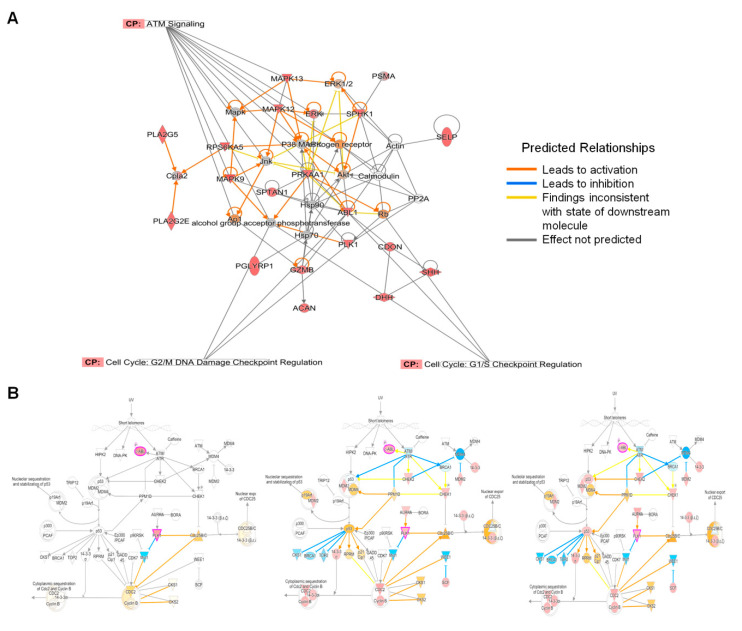
Interactions between network molecules and DNA damage checkpoint pathways. (**A**) Links between top disease and function networks at 12 hpi and immune checkpoint signaling pathways. (**B**) Changes in activation of DNA damage checkpoint signaling pathway across all three time points of (**i**) 12, (**ii**) 24 and (**iii**) 48 hpi.

IPA analyses also predicted the induction of G2/M DNA damage checkpoint regulation. Checkpoints are mechanisms that monitor various stages during cell cycle to prevent the transfer of damaged DNA to daughter cells resulting in mutation [30,31,32,33]. Three main pathways, ATM, ATR, and DNA-PK, are activated in response to DNA damage [24]. ATM signaling is activated in response to double-stranded breaks and was predicted to be activated by ZIKV at 12 hpi (Figure 4A). G2/M DNA damage checkpoint was also predicted to be induced after ZIKV infection. Induction of this checkpoint ensures that the cell cycle does not proceed to the M-Phase (mitosis) until the damaged DNA is repaired. 

IPA predicted MAPK9, MAPK12, MAPK13, ABL1 and PLK1 to be involved in the activation of ATM signaling and G2/M DNA damage checkpoint response. Key among these host proteins is ABLI, which is a tyrosine kinase present in the cytoplasm and nucleus. This protein interacts with ATM which activates several downstream molecules in response to DNA damage (Figure 4B) [34].

## 4. Discussion

We have been examining proteomic alterations induced by ZIKV in various cell types, including monkey kidney Vero [15], human U-251 astrocytoma [16] and human Sertoli [17]. Most protein dysregulation occurred at 48 hpi or later in these other cells. However, the pattern of protein dysregulation in ZIKV-infected gastrointestinal-derived human colorectal adenocarcinoma cells appears to be much more rapid, with most significantly dysregulated proteins being detected as early as 12 hpi. Another difference between these cell types is that ZIKV normally grows to substantially higher titer in Caco-2 cells than in many other cells, as previously observed [14]. It is presently unclear whether the differences in absolute virus titer produced, and more rapid kinetics of host protein dysregulation, are related. Several proteins (CXCL11, EIF5A, STAT1, CA13, ISG15, FSTL3, FN1, HIST1H1C, CST3, CTSV, PCSK9, and MDK) were similarly dysregulated in the gastrointestinal, astrocytoma, kidney and Sertoli cell types. Validation of these proteins, using other in vitro and in vivo models, may identify them as potential universal ZIKV vaccine and or antiviral targets.

Bioinformatic analyses by IPA identified several ZIKV-induced pathways which are predicted to be activated or inhibited by 12 hpi. One of the pathways was DNA damage checkpoint regulation. DNA damage checkpoints are regulatory mechanisms that exist at various stages of the cycle cell that inhibit the progression of the cell cycle when DNA damage occurs. This inhibition activates signaling pathways that initiate DNA damage repair or program the cell towards apoptosis in case the damage cannot be repaired. Some viruses, such as polyomaviruses and herpesviruses, exploit the DNA damage response to enable them to complete their replicative cycles [35,36,37,38,39]. Ataxia–telangiectasia mutated (ATM) signaling is activated in response to double-stranded DNA breaks, and was also predicted to be activated by 12 hpi. Hammack and colleagues reported that ZIKV infection activates the ATM/Chk2 signaling pathway in human neural progenitor cells and inhibits progression of cells through S phase, leading to an increase in viral replication [28]. 

Cyclin dependent kinase (CDK5) signaling, which is linked to DNA damage response, was also predicted to be inhibited by 12 hpi in the presence of ATM signaling activation. CDK5 signaling plays a significant role in neuronal function, namely the control of cytoskeletal architecture and dynamics, axonal guidance, neuronal migration, and cell adhesion, and participates in the pathological changes in neurodegenerative diseases [40]. CDK5 also plays a critical role in DNA damage response (DDR). Among the DDR, CDK5 phosphorylates ATM thereby inhibiting its kinase activity and regulating its response to double-stranded breaks that occur during the cell cycle [41,42]. The CDK5 signaling pathway was predicted to be inhibited in the presence of the activation of ATM after ZIKV infection. Inhibition of this pathway might indicate that ZIKV generally hijacks ATM signaling, as was reported in human neural progenitor cells to enhance its replication [28].

IPA also predicted several diseases and biofunctions that were altered by ZIKV infection. Among the diseases predicted after ZIKV infection was gastrointestinal diseases. ZIKV clinical symptoms are generally nonspecific; thus, ZIKV infection was often misdiagnosed in patients. ZIKV gastrointestinal involvement has not been investigated in detail yet, despite the fact patients experience gastrointestinal complications [13]. Identification of host proteins that induce gastrointestinal complications during ZIKV infection will highlight other strategies the virus adapts at the proteomic level in addition to areas which have been mainly studied. The various predicted gastrointestinal diseases were colitis, enteritis, gastroenteritis, inflammation of gastrointestinal tract, and abnormality of large intestine. All these gastrointestinal complications are predicted to be induced by most of the same dysregulated host proteins, all of which were upregulated. These included proinflammatory cytokines and cathepsins, which have been reported to be expressed in response to viral infections [43,44,45]. Some proteins that were linked to the various gastrointestinal conditions have also been reported to be involved in embryonic development. The Sonic Hedgehog (SHH) protein is critically essential for neural development [46]. This protein is important for the development of the brain and spinal cord (central nervous system), eyes, limbs, and many other parts of the body [46].

Lipid metabolism has been reported to be exploited by Flaviviruses during infection as an ATP source [47,48,49,50]. Sphingosine kinase 1 (SphK1) is a lipid kinase which is involved in various cellular functions, including proliferation, survival, tumorigenesis, development, inflammation and immunity [51,52,53]. Some of these dysregulated host proteins have been reported in other studies that utilized proteomics to identify biomarkers for the management of inflammatory bowel diseases. Periostin (POSTN) is known to bind to integrins to support adhesion and migration of epithelial cells. POSTN was significantly upregulated by ZIKV by 12 hpi as well, as reported by Chan et al., who performed a proteomic study and detected biomarkers during inflammatory bowel disease [54]. These host proteins also may serve as potential targets for the development of therapeutic intervention against ZIKV and need future validation.

Our study is the first proteomic study we are aware of to identify host proteins that ZIKV targets to induce gastrointestinal complications. We identified host proteins which, in addition to linkages to the gastrointestinal tract, are important for brain development as well. This study contributes to a better understanding of pathologies that occur during ZIKV infection.

## Figures and Tables

**Table 1:** Numbers of significantly dysregulated ZIKV-infected CaCo-2 proteins.

Number That Are Significant	Total Unique	12 Hpi	24 Hpi	48 Hpi
and fold-change > 1.000	439	252	52	150
and fold-change < 0.9999		4	0	4
and fold-change > 1.100	407	252	46	128
and fold-change < 0.9091		0	0	4
and fold-change > 1.250	261	230	23	6
and fold-change < 0.8000		0	0	3
and fold-change > 1.333	122	100	16	3
and fold-change < 0.7500		0	0	3
and fold-change > 1.375	71	52	15	2
and fold-change < 0.7273		0	0	2
and fold-change > 1.500	23	10	9	2
and fold-change < 0.6667		0	0	2
and fold-change > 2.000	2	0	2	0
and fold-change < 0.5000		0	0	0

Significance was determined by T-test and Z-score, as detailed in the materials and methods section, from three biological replicates. The 71 specific proteins dysregulated > 1.375-fold in either direction are listed in Table 2.

**Table 2:** List of significantly dysregulated Caco-2 proteins after ZIKV infection.

		12 Hpi		24 Hpi		48 Hpi	
Gene	Swissprot	Fold Change	*p*-Value	Fold Change	*p*-Value	Fold Change	*p*-Value
**Upregulated Proteins**							
EIF4G2	P78344	**2.23**	0.001	**2.02**	1.62 × 10^−5^	1.49	0.62
NTF3	P20783	**2.06**	0.005	0.81	0.50	0.90	0.04
UNC5D	Q6UXZ4	**1.93**	0.01	1.09	0.50	0.95	0.51
AK1	P00568	**1.72**	0.007	1.44	0.25	1.41	0.49
MAPK9	P45984	**1.69**	0.002	1.16	0.47	2.05	0.34
LAG3	P18627	**1.66**	0.01	1.03	0.94	1.06	0.03
NME2	P22392	**1.61**	0.02	1.36	0.21	1.16	0.13
HPX	P02790	**1.60**	0.003	1.14	0.46	0.98	0.89
FABP3	P05413	**1.56**	0.002	1.16	0.55	1.30	0.47
MAPK13	O15264	**1.51**	0.04	1.18	0.20	1.21	0.74
NTN1	O95631	**1.51**	0.03	1.16	0.54	1.08	0.09
FER	P16591	**1.51**	0.04	1.09	0.69	1.25	0.32
IL7	P13232	**1.50**	0.04	1.01	0.97	0.99	0.11
RPS6KA5	O75582	**1.49**	0.02	1.12	0.77	1.06	0.89
PPIF	P30405	**1.49**	0.01	1.20	0.34	1.03	0.96
CA13	Q8N1Q1	**1.49**	0.03	1.23	0.16	1.45	0.21
MST1	P26927	**1.48**	0.01	0.71	0.46	1.05	0.52
TIMP3	P35625	**1.48**	0.03	1.24	0.31	0.98	0.23
SHH	Q15465	**1.48**	0.04	1.10	0.68	1.13	0.94
FCGR3B	O75015	**1.46**	0.04	1.03	0.93	1.12	0.34
RSPO4	Q2I0M5	**1.46**	0.02	1.10	0.76	1.09	0.78
L1CAM	P32004	**1.44**	0.03	1.06	0.76	1.16	0.43
LCN2	P80188	**1.43**	0.048	1.17	0.22	1.11	0.74
CCL4L1	Q8NHW4	**1.43**	0.04	1.03	0.79	1.08	0.58
CA6	P23280	**1.43**	0.01	1.02	0.90	1.03	0.93
ARTN	Q5T4W7	**1.42**	0.04	1.16	0.40	1.10	0.30
TNFRSF1A	P19438	**1.42**	0.04	1.21	0.10	1.08	0.09
DPT	Q07507	**1.42**	0.03	1.11	0.50	1.09	0.25
IL3RA	P26951	**1.42**	0.047	1.21	0.36	1.11	0.44
NID1	P14543	**1.42**	0.02	1.23	0.52	0.97	0.06
GPC2	Q8N158	**1.41**	0.03	1.07	0.49	1.20	0.31
FGF7	P21781	**1.41**	0.04	1.12	0.46	1.13	0.47
TNFRSF19	Q9NS68	**1.41**	0.04	1.20	0.57	1.08	0.04
IFNL2	Q8IZJ0	**1.41**	0.01	1.16	0.48	1.20	0.21
FGF16	O43320	**1.40**	0.03	1.15	0.38	1.18	0.26
TIMP2	P16035	**1.40**	0.03	1.17	0.13	0.74	0.33
POSTN	Q15063	**1.40**	0.01	1.15	0.19	1.22	0.72
SEZ6L2	Q6UXD5	**1.40**	0.04	1.20	0.33	1.17	0.08
CHST15	Q7LFX5	**1.40**	0.02	1.05	0.80	0.94	0.65
B2M	P61769	**1.40**	0.04	1.18	0.13	0.82	0.29
ABL1	P00519	**1.39**	0.03	1.19	0.48	1.06	0.61
CST7	O76096	**1.39**	0.04	1.15	0.13	1.13	0.12
DLL4	Q9NR61	**1.39**	0.04	1.14	0.41	1.14	0.14
SIGLEC14	Q08ET2	**1.39**	0.04	1.10	0.56	1.06	0.12
MAPK12	P53778	**1.39**	0.04	1.15	0.42	1.16	0.11
BCAN	Q96GW7	**1.38**	0.02	1.34	0.33	1.12	0.39
PDE7A	Q13946	**1.38**	0.04	1.16	0.37	1.14	0.25
SPHK1	Q9NYA1	**1.38**	0.045	1.32	0.24	1.19	0.69
TIMP1	P01033	**1.38**	0.02	0.99	0.94	1.10	0.24
CFI	P05156	**1.38**	0.02	1.03	0.92	1.07	0.61
CD40LG	P29965	**1.38**	0.03	1.07	0.77	1.14	0.31
SFRP1	Q8N474	**1.38**	0.03	1.11	0.59	1.20	0.33
CSK	P41240	2.85	0.13	**2.54**	0.03	1.73	0.45
RNASEH1	O60930	2.04	0.46	**1.93**	8.44 × 10^−5^	2.32	0.22
CFL1	P23528	1.21	0.41	**1.87**	0.003	1.38	0.38
HAT1	O14929	1.34	0.48	**1.79**	0.001	1.14	0.80
SBDS	Q9Y3A5	1.63	0.12	**1.64**	0.03	1.41	0.74
WNK3	Q9BYP7	1.80	0.14	**1.58**	0.01	1.25	0.48
HK2	P52789	1.46	0.08	**1.57**	0.01	1.12	0.88
EIF4A3	P38919	1.67	0.15	**1.57**	0.02	1.27	0.60
DNAJB1	P25685	1.72	0.12	**1.56**	0.047	1.28	0.54
STAT1	P42224	1.52	0.29	**1.47**	0.008	1.43	0.24
MAP2K1	Q02750	1.42	0.20	**1.47**	0.04	1.31	0.06
MAPK8	P45983	1.79	0.09	**1.43**	0.04	1.22	0.18
IDE	P14735	1.37	0.26	**1.41**	0.006	1.29	0.27
LYZ	P61626	1.40	0.21	**1.38**	0.03	1.35	0.36
DCTPP1	Q9H773	1.64	0.19	1.51	0.08	**1.82**	0.005
IL1RL1	Q01638	2.05	0.27	1.18	0.57	**1.52**	0.02
**Downregulated Proteins**							
CTSV	O60911	1.44	0.09	1.07	0.48	**0.606**	0.04
ANG	P03950	1.44	0.34	0.92	0.54	**0.651**	0.02
FSTL3	O95633	1.28	0.18	0.87	0.67	**0.734**	0.03

Fold change cut offs used were ≥ 1.375 or ≤ 0.727, with *p*-value < 0.05. Values based on three biologic replicates. **Bolded red** represents significantly upregulated Caco-2 protein. **Bolded green** represents significantly downregulated Caco-2 protein. Non-bolded red values represent *p*-values < 0.05.
